# RGS2 squelches vascular G_i/o_ and G_q_ signaling to modulate myogenic tone and promote uterine blood flow

**DOI:** 10.14814/phy2.12692

**Published:** 2016-01-26

**Authors:** Li Jie, Elizabeth A. Owens, Lauren A. Plante, Zhuyuan Fang, Derek T. Rensing, Kevin D. Moeller, Patrick Osei‐Owusu

**Affiliations:** ^1^ Department of Pharmacology & PhysiologyDrexel University College of MedicinePhiladelphiaPennsylvania; ^2^ Nanjing University of Traditional Chinese MedicineNanjingJiangsuChina; ^3^ Department of Obstetrics & GynecologyDrexel University College of MedicinePhiladelphiaPennsylvania; ^4^ Jiangsu Hospital of Traditional Chinese MedicineNanjingJiangsuChina; ^5^ Department of ChemistryWashington UniversitySt. LouisMissouri

**Keywords:** G protein signaling, myogenic tone, calcium, RGS2, uterine blood flow

## Abstract

Uterine artery blood flow (UABF) is critical to maintaining uterine perfusion in nonpregnant states and for uteroplacental delivery of nutrients and oxygen to the fetus during pregnancy. Impaired UABF is implicated in infertility and several pregnancy complications including fetal growth restriction, small for gestational age, and preeclampsia. The etiology of abnormal UABF is not known. Here, we determined whether deficiency or loss of RGS2, a GTPase‐activating protein for G_q/11_ and G_i/o_ class G proteins, affects UABF in nonpregnant mice. We used Doppler ultrasonography to assess UABF in wild type (WT), Rgs2 heterozygous (*Rgs2+/−*), and homozygous knockout (*Rgs2−/−*) mice. Video microscopy was used for ex vivo examination of uterine artery myogenic tone and fura‐2 imaging for in vitro assessment of internal stores Ca^2+^ release. We found that baseline UABF velocity was markedly decreased while impedance measured as resistive index (WT = 0.58 ± 0.04 vs. *Rgs2−/−* = 0.71 ± 0.03, *P* < 0.01) and pulsatile index (WT = 0.90 ± 0.06 vs. *Rgs2−/−* = 1.25 ± 0.11, *P* < 0.01) was increased in *Rgs2−/−* mice. Uterine artery tone was augmented in *Rgs2+/−* and *Rgs2−/−* mice, which was normalized to WT levels following G_i/o_ and G_q_ inactivation. Conversely, blockade of ryanodine receptors increased WT myogenic tone to RGS2 mutant levels. The data together indicate that RGS2 deficiency decreases UABF by increasing myogenic tone at least partly through prolonged G protein activation. Mutations that decrease vascular RGS2 expression may be a predisposition to decreased uterine blood flow. Targeting G protein signaling therefore might improve uterine and uteroplacental underperfusion disorders.

## Introduction

Small caliber blood vessels constrict and relax in response to changes in intraluminal pressure. Increases in intraluminal pressure cause vasoconstriction; conversely, decreases in intraluminal pressure have the opposite effect of causing vasodilatation. This regulatory mechanism, known as myogenic response, is a vascular smooth muscle‐intrinsic property and a component of autoregulatory mechanisms that enable small blood vessels, including feed arteries and arterioles, to maintain nearly constant organ blood flow in the face of moment‐to‐moment fluctuations in systemic arterial pressure.

The uterine artery is a primary feed artery that conducts oxygenated blood and nutrients from the iliac artery to the uterine vascular bed. Proper uterine artery blood flow is essential for fetal implantation following fertilization, and impaired flow via this artery is implicated in unexplained recurrent miscarriages (Steer et al. 1995; Cacciatore et al. 1996; Salle et al. 1998). A particularly notable anatomical and adaptive feature of the uterine vascular bed is that collateral blood supply to the myometrium – and the placenta during pregnancy – is conducted by arcuate and radial arteries that emanate from the uterine artery (Osol and Mandala 2009). During pregnancy, endovascular trophoblast invasion widens and converts radial arteries to capacitance vessels with little or no resistance to blood flow to the placenta (Thaler et al. 1990; Osol and Mandala 2009). Thus, lumen diameter of the uterine artery becomes a primary determinant of uterine blood flow during pregnancy. Therefore, control mechanisms that regulate uterine artery myogenic tone and vessel diameter, are critical to maintaining appropriate uterine perfusion under all physiological and pathological states.

The mechanisms that regulate uterine vascular tone and myogenic response are poorly understood. Nonetheless, several studies have established that myogenic constriction is initiated when mechanosensors in the smooth muscle layer detect changes in intramural pressure and activate signaling cascades that result in a rise in intracellular Ca^2+^ concentration in vascular smooth muscle cells (VSMC) (Davis and Hill 1999; Hill and Meininger 2012). It is also broadly accepted that myogenic constriction does not require the release of endogenous substances or neuronal input (Davis and Hill 1999). However, myogenic tone can be modulated by receptor‐independent activation of heterotrimeric G proteins via signaling mechanisms (Kauffenstein et al. 2012). We therefore hypothesized that targeted mutations that decrease or abolish the expression of proteins that regulate G protein signaling would lead to decreased uterine artery blood flow due to increased and/or prolonged G protein activation and sustained myogenic constriction.

The kinetics and strength of G protein activity are tightly regulated by a family of RGS (regulators of G protein signaling) proteins (Ross and Wilkie 2000). RGS proteins act as GTPase activating proteins (GAPS) that accelerate GTP hydrolysis by the intrinsic GTPase activity of heterotrimeric G proteins’ alpha subunit, thereby accelerating the rate of termination of G protein activation (Watson et al. 1996). Besides their GAP function, RGS proteins can regulate signaling via non‐GAP domains present in certain RGS families (Nguyen et al. 2009; Sethakorn et al. 2010). Mutations that decrease the expression and/or function of RGS proteins are linked to human diseases such as hypertension, preeclampsia, and vision impairment (Gu et al. 2008; Martemyanov and Arshavsky 2009; Kvehaugen et al. 2014). Among more than 30 mammalian RGS proteins discovered to date, several members of the R4/B RGS family, including RGS2‐5, 8, 13, 16, 18, and 21, have been shown to play a prominent role in the cardiovascular system (Chidiac and Roy 2003; Riddle et al. 2005; Gu et al. 2009). Among the R4 RGS proteins, RGS2 and 5 are implicated in gestational hypertension and preeclampsia (Kvehaugen et al. 2013; Holobotovskyy et al. 2015). RGS2 has been shown to have a more potent GAP function toward G_q_ (Heximer et al. 1997; Heximer 2004), the primary G protein that stimulates PLC‐mediated rise in intracellular Ca^2+^ for initiating VSMC contraction and for the development of myogenic tone (Davis and Hill 1999). Recent studies have shown that a single‐nucleotide polymorphism (SNP) of human *Rgs2* gene (rs4606) is a risk modifier for the development and progression of preeclampsia (Kvehaugen et al. 2013). Rs4606, is a 3′ untranslated region (3′UTR) C1114G polymorphism linked to decreased RGS2 expression and hypertension (Semplicini et al. 2006). In primary cells obtained from individuals harboring the *Rgs2* SNP, angiotensin II‐induced Ca^2+^ mobilization was enhanced, correlating with decreased RGS2 mRNA expression in these cells (Semplicini et al. 2006).

Here, we have examined nonpregnant mice lacking one or both copies of functional *Rgs2* gene to determine whether RGS2 protein is required for maintaining normal uterine artery blood flow and myogenic tone. Our noninvasive in vivo analysis combined with ex vivo and in vitro studies of mouse uterine arteries, and isolated VSMCs show that loss of just one copy of *Rgs2* is sufficient to impair G protein regulation leading to augmented uterine artery myogenic tone and decreased uterine artery blood flow.

## Materials and Methods

### Animal preparation

Studies were performed in accordance with protocols approved by the Animal Studies Committee of Drexel University College of Medicine in accordance with the National Institutes of Health Guidance for the Care and Use of Laboratory Animals. All experiments were performed using 8‐ to 12‐week‐old female mice that have been backcrossed extensively into the C57BL/6 background (Charles River). Generation of *Rgs2+/−* and *Rgs2−/−* mice has been described previously (Oliveira‐Dos‐Santos et al. 2000). Mice were provided access to food and water ad libitum in our institution's animal facility at 22°C and a 12‐h light/dark cycle.

### Uterine blood flow assessment by Doppler ultrasound

Twenty‐four hours prior to ultrasound examination, mice were placed under isoflurane anesthesia (2–3% isoflurane; Baxter Healthcare Corporation, Deerfield, IL, plus 1.5 L/min O_2_) to shave off hair from the abdomen with clippers and hair removal gel. The next day, mice were maintained under light anesthesia (1–1.5% isoflurane plus 1.5 L/min O_2_) on a heating platform (Vevo 2100 Imaging Station; Visual Sonics Inc. Toronto, Ontario, Canada) and gently secured with adhesive tape. Body temperature was maintained at 37°C while heart rate was recorded for the entirety of the ultrasound to ensure the mice remained within safe physiological limits. Uterine artery blood flow was measured by the Doppler waveforms recorded using a 400 MHz probe placed over the lower abdomen covered with coupling gel, as previously described (Hernandez‐Andrade et al. 2014). The Doppler probe was held in a fixed position and mobilized by a holding stand. Ultrasound recordings were taken from the right and left uterine arteries close to the bladder at a 30° angle of insonation. Three waveforms were recorded from each side of the uterine horns using a Doppler gate size and sensitivity set to 3 and 5, respectively. After acquisition of the waveforms, the mice were removed from the isoflurane anesthesia, returned to their home cages, and used later for ex vivo uterine artery experiments.

From each acquired waveform, peak systolic velocity (PSV), least diastolic velocity (LDV), and mean velocity (MV) were calculated. The average PSV, LDV, and MV for each mouse was calculated and used to derive the following indices: Resistive Index, (RI) = (*V*_max_ − *V*_min_)/*V*_max_; Pulsatile Index, (PI) = (*V*_max_ − *V*_min_)/*V*_mean_; and PSV/LDV ratio = *V*_max_/*V*_min_; where *V*_max_ = PSV, *V*_min_ = LDV and *V*_mean_ = MV.

### Determination of uterine artery myogenic tone

To determine the effects of RGS2 deficiency on uterine artery blood flow, we performed structural–functional analysis of uterine arteries using Living Systems vessel chamber (Catamount, Burlington, VT) and IonOptix vessel dimensions analysis software as previously described (Sweazea and Walker 2012). Briefly, mice were deeply anesthetized with ketamine/xylazine (ketamine; 43 mg/kg, i.p., and xylazine; 6 mg/kg, i.p.) and killed by cervical dislocation. The uterus was harvested and placed in cold physiological saline solution (PSS) of the following composition: (in mmol/L) 140 NaCl, 5 KC1, 1.2 MgSO_4_, 2.0 CaC1_2_, 10 NaAcetate, 10 HEPES, 1.2 Na_2_H_2_PO_4_, 5 glucose, and pH adjusted to 7.4 with NaOH. Segments of the main uterine arteries were isolated, excised, and transferred into a vessel chamber, where they were cannulated at both ends with glass pipettes and secured with nylon ligature. Intraluminal pressure and vessel bath temperature were maintained by servo‐controlled pressure pump and temperature control systems, respectively. The vessel lumen and chamber were filled with PSS of the same composition described above. Following a 30‐min equilibration period at 37°C and 60 mmHg, cannulated vessels were treated with increasing concentrations of potassium chloride (KCl)‐PSS to assess viability. Normally, viable vessels developed spontaneous tone during wash period following high K^+^ treatment. Therefore, any vessel with less than 50% reduction in lumen diameter at the highest K^+^ concentration (80 mmol/L) and unable to develop spontaneous tone was excluded from the experiment. To generate pressure–diameter curves, cannulated uterine arteries were subjected to stepwise increases in intraluminal pressure from 10 to 140 mmHg (20 mmHg increments from 20 mmHg, 5 min each). For experiments involving pharmacological agents, vessels were incubated with drugs after the initial pressure steps, followed by another pressure steps in the presence of the drug. Then, the arteries were equilibrated at 60 mmHg for 30 min in a Ca^2+^‐free PSS containing 3 mmol/L EGTA, after which the pressure steps protocol was applied again to determine passive diameters. In cases where more than one uterine artery segment from one mouse was used, we calculated the average and included as *n* of 1. Mechanical properties of uterine arteries from wild type, *Rgs2+/−* and *Rgs2−/−* mice were analyzed by calculating wall tension, circumferential wall strain, circumferential wall stress, and incremental distensibility, using measured values of lumen diameter and vessel wall thickness obtained from pressure‐step protocol performed after treating the vessels with Ca^2+^‐free PSS containing EGTA, as described above. Functional properties of uterine arteries were assessed by determining active constriction and myogenic response to step increases in intraluminal pressure. Wall tension in the presence of Ca^2+^ was calculated to determine the effectiveness of myogenic response in reducing wall strain following step increases in transmural pressure.

### Calcium imaging

Imaging of Ca^2+^ signaling in freshly isolated uterine artery smooth muscle cells was performed as previously described (Osei‐Owusu et al. 2007). Briefly, the uterus from 8‐ to 12‐week‐old wild‐type and *Rgs2−/−* female mice was harvested as described, and placed in cold (4°C) PSS. Following isolation and removal of tissue fat, the uterine arteries from both uterine horns were cut into small ~1 ‐mm pieces, and placed in a vial of cold PSS on ice. For each experiment, vessels from two mice of the same genotype were pooled to provide enough cells for Ca^2+^ imaging. The vessels were incubated in PSS containing papain (0.3 mg/ml) and dithiothreitol (1.0 mg/mL) at 37°C for 30 min. The incubation solution was then changed to PSS containing collagenases F (0.3 mg/mL) and H (0.7 mg/mL) and incubated at 37°C for 20 min. The vessels were gently washed three times with prewarmed Ca^2+^‐free PSS. A final volume of 200 *μ*L of Ca^2+^‐free PSS was added to the vessels and triturated with increasingly smaller glass pipets to disperse smooth muscle cells from the vessel wall. The dispersed cells were seeded on glass coverslips coated with CellTak and then loaded with fura‐2 AM (4 μmol/L) in PSS containing 1% Pluronic F‐127 (Life Technologies), by incubating at room temperature in the dark for 30 min. Afterwards, the cells were washed several times with PSS and incubated at 37°C in the dark for another 30 min to allow fura‐2 AM de‐esterification. Coverslips containing isolated smooth muscle cells were mounted on an Olympus inverted microscope equipped with a CCD camera (Hamamatsu ORCA‐03G, Tokyo, Japan). The Cells were continuously perfused with PSS at room temperature. To remove extracellular Ca^2+^, cells were perfused with Ca^2+^‐free PSS containing 3 mmol/L EGTA for 5 min. Next, Ca^2+^ release from internal stores was stimulated by perfusing the cells with Ca^2+^‐free PSS containing 5 μmol/L ionomycin for 5 min. In another set of experiments, the cells were perfused with PSS containing ryanodine (10 μmol/L) for 5 min, followed by perfusion with Ca^2+^‐free PSS and ionomycin treatment as described above, all in the presence of the same concentration of ryanodine. Throughout the experimental protocol, fura‐2 fluorescence images were acquired at 3‐sec intervals and analyzed using the software MetaFluor 7.7.9 (Molecular Devices, Sunnyvale, CA). After background correction, image ratios were calculated with region of interest (ROI) representing one cell. The fluorescence ratio of each ROI was calculated as fluorescence intensity at 340 nm to fluorescence intensity at 380 nm. All drug treatments were perfused through the coverslip chamber.

### Pharmacological agents

Pertussis toxin (PTX, 200 ng/mL; Sigma), an ADP ribosylating agent, was used to specifically inactivate G_i/o_ class G proteins. WU‐07047 (20 μmol/L; Washington University) was used to inhibit G_q_ class G proteins. Thapsigargin (1 μmol/L; Tocris Biosciences) was used to block sarcoplasmic reticulum (SR) Ca^2+^ uptake via SERCA pump. Ryanodine (10 μmol/L; Tocris Biosciences) was used to target ryanodine receptors (RyRs). N,N,N′,N′‐tetrakis(2‐pyridylmethyl)ethane‐1,2‐diamine (TPEN, 50 μmol/L; Sigma) was used to sequester Ca^2+^ in intracellular stores. Ionomycin (5 μmol/L) was used to stimulate Ca^2+^ release from internal stores.

### Statistical analysis

All data are mean ± standard error. Baseline parameters were analyzed by unpaired Student's *t*‐test. For assessment of within‐group effects of various drug treatments on myogenic tone, one‐way ANOVA with repeated measures was used, and two‐way ANOVA with repeated measures was used for between‐group comparisons with Student–Newman–Keuls post hoc tests. The level of significance was set at *P *<* *0.05.

## Results

### Decreased uterine blood flow and increased resistivity in RGS2 deficient mice

**Figure 1:**
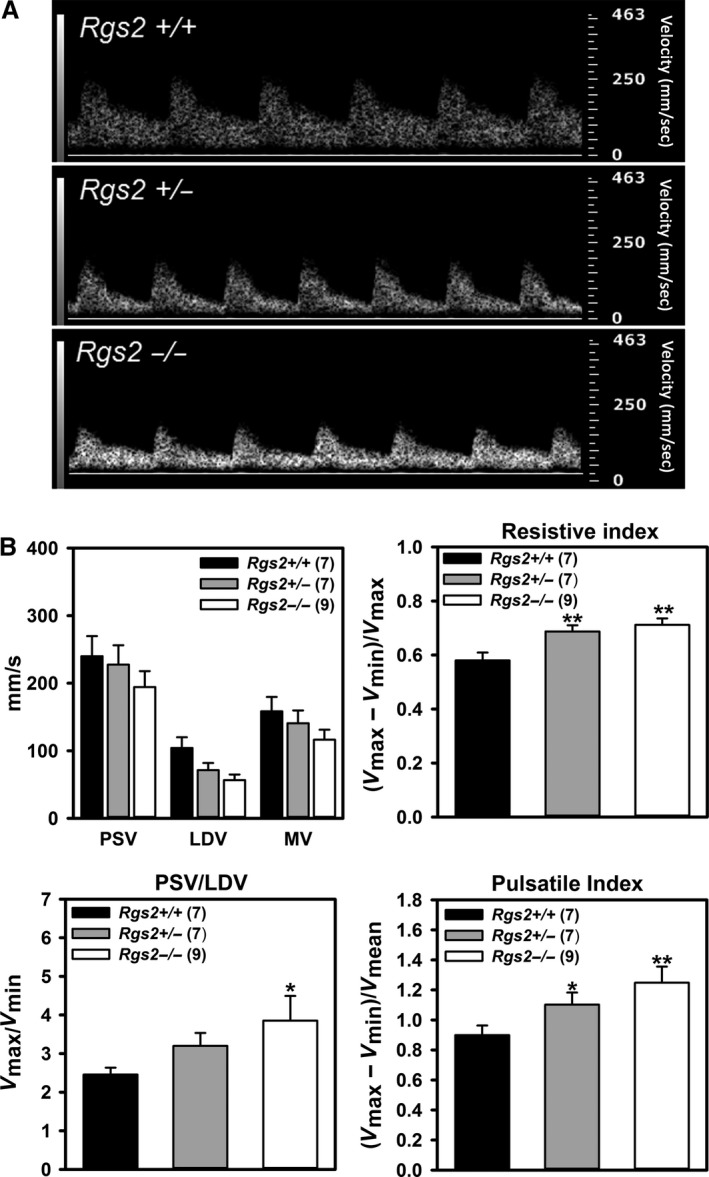
RGS2 deficiency increases impedance to uterine artery blood flow. (A) Representative uterine artery Doppler ultrasonography waveforms from nonpregnant wild‐type (*Rgs2+/+*), and Rgs2 heterozygous (*Rgs2+/−*) and homozygous knockout (*Rgs2−/−*) mice obtained under isoflurane anesthesia. Pulse wave velocities were acquired at an angle of 30° and frequency of 24 Hz. (B) Uterine artery waveforms from *Rgs2+/+* (*n* = 7), *Rgs2+/−* (*n* = 7) and *Rgs2−/−* (*n* = 9) were analyzed to obtain peak systolic velocity (PSV or *V*_max_), least diastolic velocity (LDV or *V*_min_), and mean velocity (MV or *V*_mean_), which were used to derive resistive index, pulsatile index, and PSV‐to‐LDV ratio for each genotype. Values are mean ± SE. *^,^**P <* *0.05, 0.01 versus *Rgs2*+/+.

Previous studies showed that loss of RGS2 increases blood pressure and decreases renal blood flow (Heximer et al. 2003; Osei‐Owusu et al. 2015). Therefore, we determined whether RGS2 deficiency affects uterine artery blood flow. We used uterine Doppler ultrasonography to assess uterine artery hemodynamics and waveforms in nonpregnant wild type (WT), *Rgs2+/−* and *Rgs2−/−* mice. Uterine artery blood flow waveforms were similar in all groups (Fig. 1A). As shown in Figure 1B, peak PSV, LDV, and MV trended lower from WT to *Rgs2−/−* mice. Moreover, all indices of uterine artery flow impedance, including PSV‐to‐LDV ratio, RI, and PI were significantly increased in both *Rgs2+/−* and *Rgs2−/−* relative to WT mice. Together, these data indicated that impedance to uterine artery blood flow increases with decreasing expression of RGS2 in nonpregnant mice.

### RGS2 deficiency increases uterine artery myogenic response

**Figure 2:**
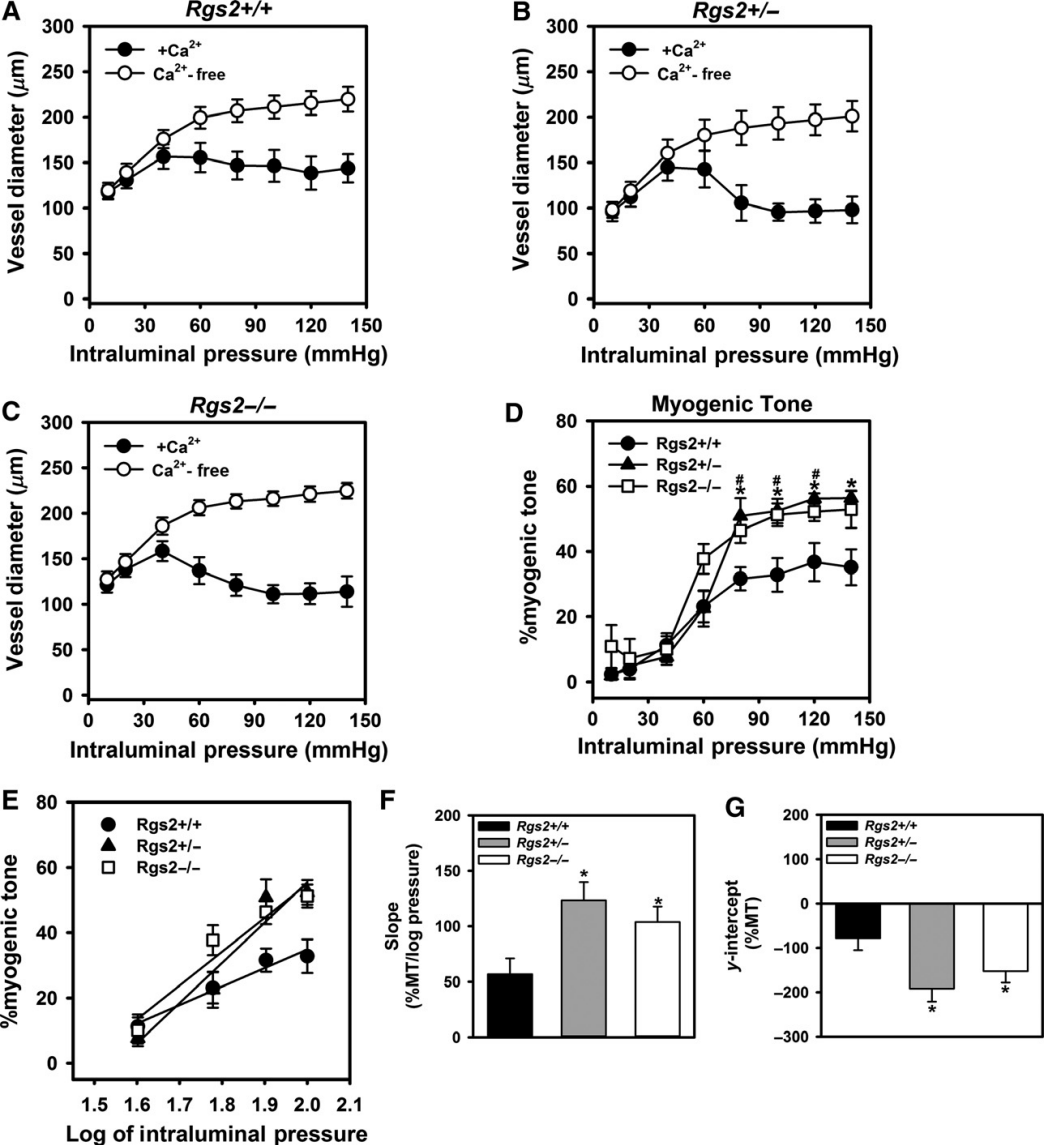
RGS2 deficiency increases uterine artery resting myogenic tone and sensitivity of myogenic response to increases in intraluminal pressure. (A–C) Uterine artery diameter of wild type (A, *Rgs2+/+*), *Rgs2* heterozygous (B, *Rgs2+/−*), and homozygous knockout (C, *Rgs2−/−*) mice were measured at increasing intraluminal pressure in the presence (closed circles) and absence (open circles) of Ca^2+^ in the superfusing physiological saline solution (PSS). (D) Percent myogenic tone at each intraluminal pressure calculated using vessel diameters measured in the presence (active diameter) and absence (passive diameter) of extracellular Ca^2+^. (E) Percent myogenic tone values in D re‐plotted with the *x*‐axis transformed to a log scale to generate a linear intraluminal pressure – %myogenic tone relationship. The corresponding slopes and *y*‐intercepts of the linear plots in E are plotted in F and G, respectively. Values are mean ± SE. *^,#^ *P *<* *0.05, versus *Rgs2*+/+.

To determine the mechanism mediating decreased uterine artery blood flow in nonpregnant RGS2‐deficient mice, we examined uterine artery myogenic response to increasing intraluminal pressure using isolated vessel preparation approach. Figure 2A–G show changes in inner vessel diameter and myogenic tone in response to step increases in intraluminal pressure in the presence and absence of Ca^2+^ in uterine arteries from WT, *Rgs2+/−*, and *Rgs2−/−* mice. Under Ca^2+^‐free conditions, the diameter of uterine arteries from all groups increased equally with increasing intraluminal pressure (Fig. 2A–C, open circles). In contrast, vessel diameter began to decrease when intraluminal pressure was increased from 40 to 60 mmHg (closed circles), indicating pressure‐induced vasoconstriction. Uterine arteries from all groups elicited a contractile response, which was exaggerated in arteries from *Rgs2+/−* and *Rgs2−/−* mice (Fig. 2B and C). The pressure‐induced contractile responses translated to pronounced myogenic response in uterine arteries from *Rgs2+/−* and *Rgs2−/−* mice (Fig. 2D). At 80 mmHg of intraluminal pressure, myogenic tone was significantly lower in WT relative to *Rgs2+/−* and *Rgs2−/−* (WT = 31.6 ± 3.6 vs. *Rgs2+/−* = 50.8 ± 4.9 vs. *Rgs2−/−* = 50.0 ± 4.8) and remained elevated at all intraluminal pressures. Next, we determined whether RGS2 deficiency affected uterine artery myogenic response sensitivity and resting myogenic tone. We determined sensitivity by calculating the slope of the linear line of best fit of myogenic tone at 40, 60, 80, and 100 mmHg in all groups (Fig. 2E). The same line of best fit was used to determine resting myogenic tone, indicated by the *y*‐intercept. As shown in Figure 2F and G, RGS2 deficiency increased the sensitivity of uterine artery myogenic response, indicated by increased slope of the myogenic tone–log of intraluminal pressure linear curve. Similarly, when compared to WT, uterine arteries from *Rgs2+/−* and *Rgs2−/−* mice showed increased resting myogenic tone indicated by a more negative *y*‐intercept (Fig. 2G). Because RGS2 deficiency in the vasculature causes impaired endothelium‐dependent vasodilation in mesenteric arteries (Osei‐Owusu et al. 2012), we determined whether increased uterine artery myogenic tone could be due partly to loss of RGS2 in uterine artery endothelium. Stimulation of uterine arteries with increasing concentrations of acetylcholine caused similar vasodilatory responses in uterine arteries from WT, *Rgs2+/−*, and *Rgs2−/−* mice (data not shown), indicating the absence of endothelial dysfunction in the augmented uterine artery myogenic tone in *Rgs2−/−* mice.

### Effects of RGS2 deficiency on wall tension

**Figure 3:**
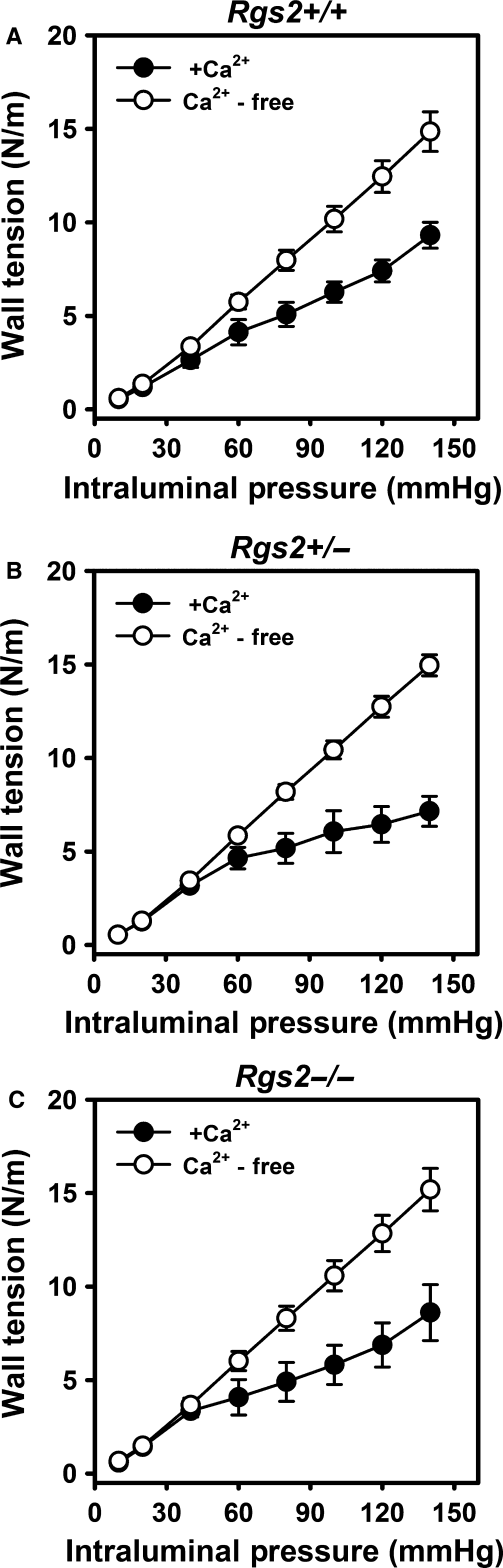
RGS2 deficiency does not affect changes in wall tension in response to increasing intraluminal pressure in uterine arteries. Active (closed circles) and passive (open circles) wall tension in uterine arteries from wild type (A, *Rgs2+/+*), *Rgs2* heterozygous (B, *Rgs2+/−*), and homozygous knockout (C, *Rgs2−/−*) mice in response to increases in intraluminal pressure. In all genotypes, increasing intraluminal pressure in the presence of Ca^2+^ induced a myogenic response that significantly reduced wall tension from 60 to 140 mmHg. Values are mean ± SE.

Figure 3A–C show the development of wall tension in uterine arteries with increasing intraluminal pressure of WT, *Rgs2+/−* and *Rgs2−/−* mice, respectively. In the absence of Ca^2+^, uterine arteries from all groups similarly developed increasing wall tension with increasing intraluminal pressure (open circles). In the presence of Ca^2+^, wall tension decreased with increasing intraluminal pressure from 60 mmHg in all groups (closed circles). At 80 mmHg, wall tension was lower in uterine arteries from *Rgs2+/−* relative to WT mice (WT = 4.6 ± 0.8 vs. *Rgs2+/−* = 3.9 ± 0.0); however, the difference did not reach statistical significance (*P *=* *0.18).

### RGS2 deficiency increases myogenic tone without altering mechanical properties of uterine arteries

**Figure 4:**
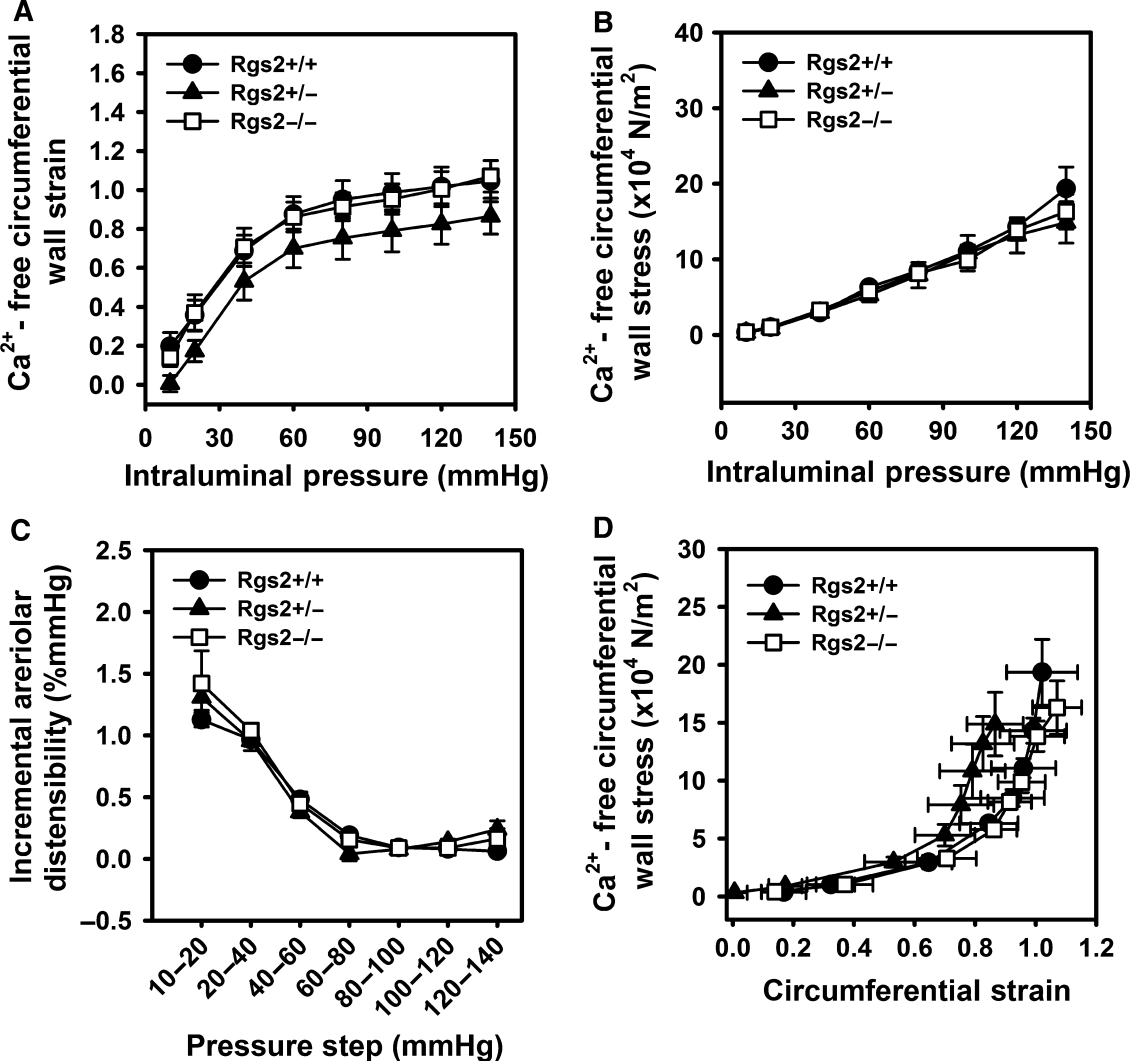
Mechanical properties of uterine arteries are not affected by RGS2 deficiency. Circumferential wall strain (A) and wall stress (B), and incremental arteriolar distensibility of uterine arteries from wild type (*Rgs2+/+,* closed circle), *Rgs2* heterozygous (*Rgs2+/−*, closed triangle), and homozygous knockout (*Rgs2−/−*, open square) mice were determined under Ca^2+^‐free conditions with increasing intraluminal pressure. (D) Circumferential wall stress‐strain relationship generated using values from figures A and B. Values are mean ± SE.

To determine whether altered mechanical properties contributes to increased myogenic tone in uterine arteries from RGS2‐deficient mice, we examined wall strain, stress, incremental distensibility, and stress–strain relationship under Ca^2+^‐free, passive conditions. As shown in Figure 4A–D, mechanical properties of uterine arteries were not affected by RGS2 deficiency. Moreover, average wall thickness, cross‐sectional area, lumen diameter, and media‐to‐lumen ratio were not changed by RGS2 deficiency (Table 1).

**Table 1:** Morphometric data of pressurized uterine arteries from *Rgs2+/+, Rgs2+/−*, and *Rgs2−/−* mice at 80 mmHg in Ca^2+^‐free PSS

Parameter	*Rgs2+/+*	*Rgs2+/−*	*Rgs2−/−*
No. of arteries	14	7	7
Average wall thickness, *μ*m	15.1 ± 6.3	13 ± 5.69	12.7 ± 5.1
Lumen diameter, *μ*m	203 ± 45	184 ± 29	201 ± 40
Cross sectional area, ×1,000 *μ*m^2^	10.2 ± 3.9	7.6 ± 2.7	8.3 ± 2.6
Media/lumen ratio	0.16 ± 0.12	0.15 ± 0.09	0.14 ± 0.09

Data are means ± s.e. *Rgs2+/+*, mice with wild‐type *Rgs2*;* Rgs2+/−*,* Rgs2* heterozygous knockout mice; *Rgs2−/−*, homozygous knockout mice.

### Increased signaling via G_q_ and G_i/o_ class G proteins contributes to augmented uterine artery myogenic tone in RGS2 deficient mice

**Figure 5:**
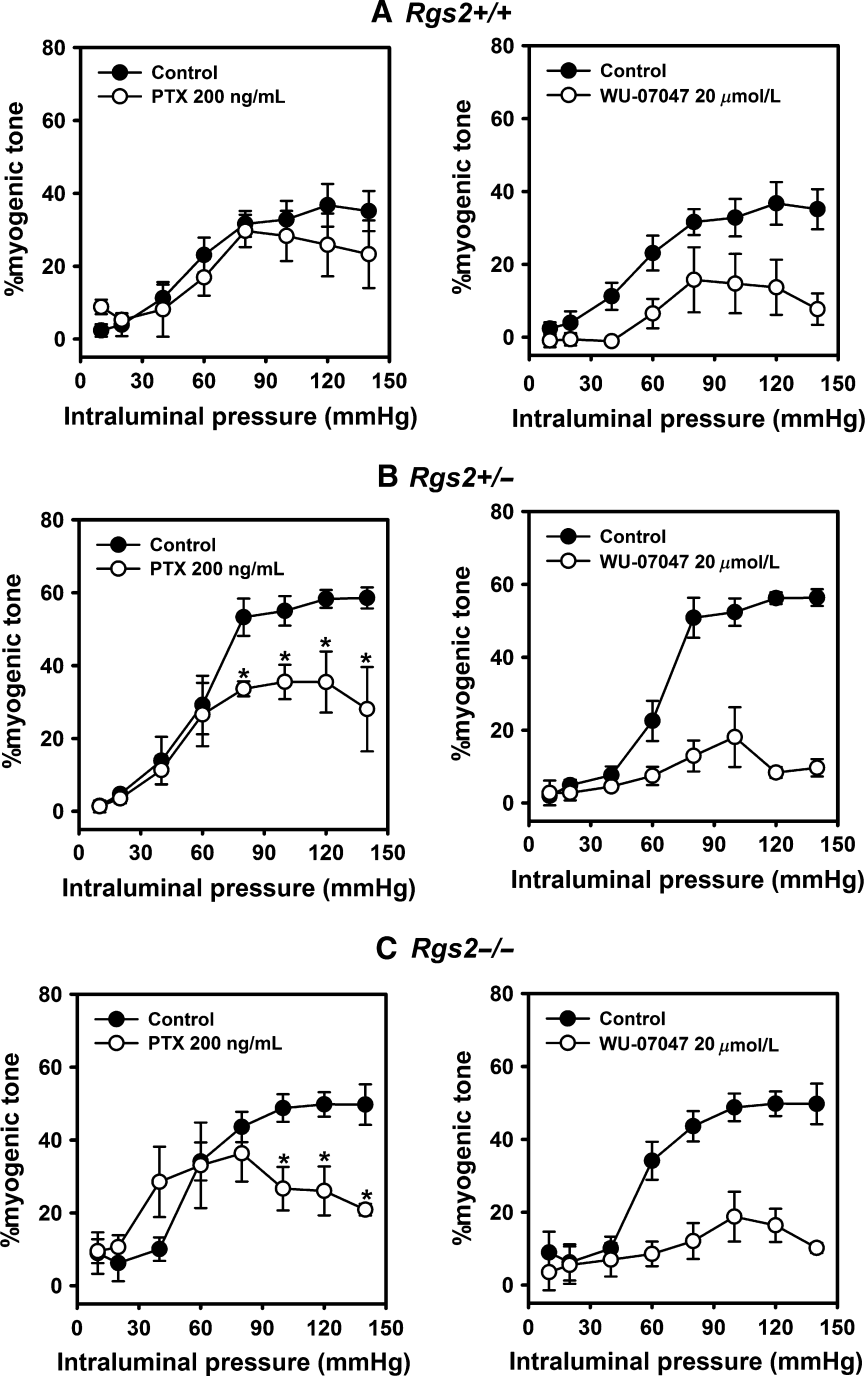
Increased signaling via G_i/o_ and G_q_ class G proteins contributes to augmented myogenic tone in uterine arteries from RGS2‐deficient mice. Myogenic response of uterine arteries from wild type (A, *Rgs2+/+*), and *Rgs2* heterozygous (B, *Rgs2+/−*) and homozygous knockout (C, *Rgs2−/−*) mice in the absence (closed circle) and presence (open circle) of G_i/o_ inhibitor, pertussis toxin (PTX, left panel) and G_q_ inhibitor, WU‐07047 (right panel). PTX reduced myogenic tone in uterine arteries from *Rgs2+/−* and *Rgs2−/−* mice to *Rgs2+/+* control levels while it had no effect on myogenic tone in arteries from *Rgs2+/+* mice. In contrast, WU‐07047 reduced myogenic tone in all genotypes to similar levels, with a greater change in uterine arteries from *Rgs2+/−* and *Rgs2−/−* mice.

Several reasons led us to determine whether increased G protein signaling contributes to augmented myogenic tone in uterine arteries from RGS2‐deficient mice. First, signaling via heterotrimeric G proteins are known to play critical roles in myogenic tone and contractile mechanisms in vascular smooth muscle (Davis and Hill 1999; Wirth et al. 2008; Kauffenstein et al. 2012). Second, RGS2 is known to be a potent GAP for G_q_ and G_i/o_ class G proteins (Heximer et al. 1997; Osei‐Owusu et al. 2012). Third, loss of RGS2 leads to augmented GPCR‐induced Ca^2+^ transients in VSMCs and prolonged vasoconstrictor responses in mice (Heximer et al. 2003; Tang et al. 2003; Osei‐Owusu et al. 2007). Therefore, to determine whether increased signaling via G_i/o_ class plays a role in augmented myogenic tone, we examined the effects of inhibiting G_i/o_ class G proteins with pertussis toxin (PTX) on pressure‐induced myogenic response in uterine arteries. As shown in Figure 5A left panel, PTX had no effect on myogenic response in uterine arteries from WT mice. In contrast, the same concentration of PTX decreased myogenic tone in uterine arteries from *Rgs2+/−* and *Rgs2−/−* mice (Fig. 5B and C, left panel). At 80 mmHg, PTX treatment reduced myogenic tone in both *Rgs2+/−* and *Rgs2−/−* to WT control level (WT con = 31.6 ± 3.6 vs. *Rgs2+/−* = 33.6 ± 2.1 vs. *Rgs2−/−* = 33.2 ± 10).

Next, we determined whether increased uterine artery myogenic tone in RGS2 deficiency also involves augmented signaling via G_q_ class G proteins. As shown in Figure 5A–C, right panels, treatment of uterine arteries with the novel G_q_ inhibitor, WU‐07047(Rensing et al. 2015), reduced myogenic response to similar levels at all intraluminal pressures in all genotypes. The effect of WU‐07047 on myogenic tone was more pronounced in arteries from *Rgs2+/−*and *Rgs2−/−* mice (Fig. 5B and C). These results together indicated that G protein signaling regulation by RGS2 plays a central role in modulating myogenic tone development in uterine arteries.

### RGS2 regulates myogenic tone by modulating G protein‐mediated Ca^2+^ release from internal stores

**Figure 6:**
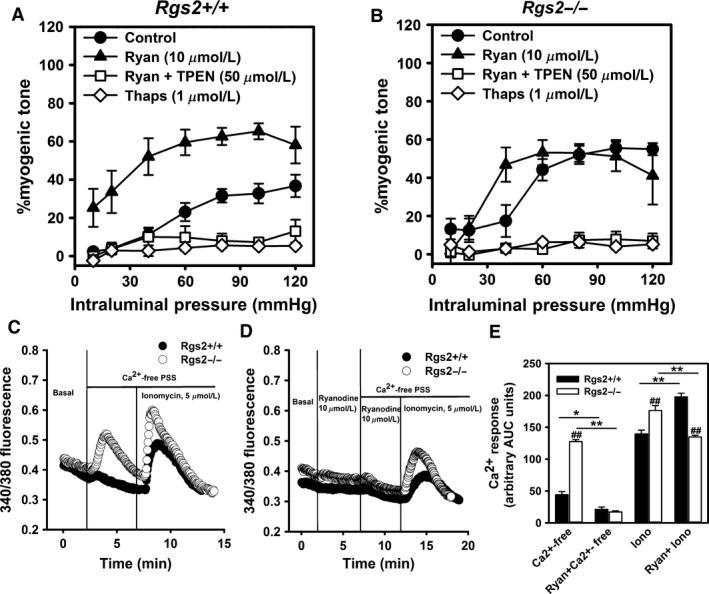
Increased Ca^2+^ release from internal stores mediates augmented myogenic tone in RGS2 deficient mice. (A & B) Myogenic response of uterine arteries from wild type (*Rgs2+/+*) and Rgs2 knockout (*Rgs2−/−*) mice in the absence and presence of ryanodine (Ryan, closed triangles), thapsigargin (Thaps, open diamonds), or Ryan plus N,N,N′,N′‐tetrakis(2‐pyridylmethyl)ethane‐1,2‐diamine (Ryan + TPEN, open squares). Thaps and Ryan + TPEN almost completely blocked myogenic response in both genotypes. (C) Depletion of extracellular Ca^2+^ with Ca^2+^‐free PSS induces robust Ca^2+^ release from internal stores in uterine artery smooth muscle cells (SMCs) from *Rgs2* knockout (*Rgs2−/−*, open circles) relative and wild‐type (*Rgs2+/+*, closed circles) mice. Ionomycin induces Ca^2+^ release from internal stores in SMCs from both genotypes. (D) Ca^2+^‐free PSS but not ionomycin‐induced Ca^2+^ release from internal stores is almost completely abolished by Ryan in SMCs from *Rgs2−/−* mice. (E) A plot of area under the curve (AUC) of fura‐2 fluorescence shown in C and D. Note the augmentation of ionomycin‐induced Ca^2+^ response by Ryan in *Rgs2+/+* SMCs, which correlates with enhanced myogenic response in *Rgs2+/+* uterine arteries in the presence of Ryan in A. Values are mean ± SE. *^,^**P <* *0.05, 0.01; ^##^ *P *<* *0.01 versus corresponding *Rgs2+/+*.

Increased cytosolic Ca^2+^ concentration due to influx from extracellular milieu and release from internal stores is required for myogenic response. In VSMCs, RGS2 regulates G protein–phospholipase C signaling pathway that mediates GPCR‐evoked cytosolic Ca^2+^ rise and subsequent contraction (Heximer et al. 2003; Gu et al. 2008). Therefore, we determined whether in the absence of ligand‐dependent GPCR activation, this signaling axis also contributes to augmented myogenic response due to RGS2 deficiency. Figure 6 shows myogenic response of uterine arteries from WT and *Rgs2−/−* mice in the absence and presence of the ryanodine receptor (RyR) inhibitor, ryanodine, sarco/endoplasmic reticulum Ca^2+^‐ATPase (SERCA) inhibitor and internal Ca^2+^ depleting agent, thapsigargin, and internal stores Ca^2+^ chelator, TPEN. In the presence of thapsigargin, myogenic response was almost completely abolished in both groups. In contrast, ryanodine treatment increased myogenic response in WT arteries to *Rgs2−/−* level from 40 to 140 mmHg intraluminal pressures (Fig. 6A). In *Rgs2−/−* arteries, ryanodine significantly increased myogenic tone only at 40 mmHg, compared to *Rgs2−/−* control (Fig. 6B). In both groups, the effect of ryanodine on myogenic tone was almost completely abolished when co‐incubated with TPEN to chelate Ca^2+^ in internal stores (Fig. 6A and B). Indeed, the myogenic response following ryanodine‐plus‐TPEN treatment was similar to the effect of thapsigargin in both groups. Together, these results suggested that increased myogenic tone due to RGS2 deficiency is mediated at least partly by increased Ca^2+^ release from internal stores and inhibition of RyR activity.

### RGS2 regulates ryanodine receptor‐mediated Ca^2+^ release from internal stores in uterine artery smooth muscle cells

Next, we examined cytosolic Ca^2+^ in vitro to determine how the absence of RGS2 affects Ca^2+^ retention and release from internal stores using fura‐2 imaging of freshly isolated uterine artery smooth muscle cells (SMCs). As shown in Figure 6C, removal of extracellular Ca^2+^ caused a slight increase in fura‐2 fluorescence ratio in WT SMCs. In contrast, the absence of extracellular Ca^2+^ induced a marked increase in fura‐2 fluorescence ratio in SMCs from *Rgs2−/−* mice. In both groups of cells, subsequent treatment with ionomycin in Ca^2+^‐free PSS induced a robust increase in fluorescence signal (Fig. 6C and E). The fluorescent signal triggered by Ca^2+^‐free PSS in *Rgs2−/−* SMCs was almost completely abolished when the cells were pretreated with ryanodine (Fig. 6D and E). In the presence of ryanodine, total ionomycin‐induced fura‐2 fluorescent signal was enhanced in WT while it was attenuated in *Rgs2−*/*−* SMCs (Fig. 6E). Together, these data indicated that releasable Ca^2+^ pool from internal stores is increased in the absence of RGS2. The data also indicated that RGS2 regulates RyR‐mediated Ca^2+^ release from internal stores.

## Discussion

The main finding in this study is that the effects of G protein signaling on uterine artery myogenic tone must be tightly controlled by RGS2, a GTPase activating protein that regulates the kinetics and amplitude of G_q_ and G_i/o_ class G proteins, to maintain normal uterine artery blood flow. We reached this conclusion by analyzing uterine artery blood flow and myogenic tone in nonpregnant WT mice and congenic *Rgs2+/−* (thus making half the amount of functional RGS2 protein) and *Rgs2−/−* mutants. In vivo*,* RGS2 deficiency markedly decreases uterine artery blood flow by increasing impedance; ex vivo, RGS2 deficiency increases myogenic contractile response to increasing intraluminal pressure in uterine arteries; and in vitro, RGS2 deficiency increases Ca^2+^ release from internal stores via ryanodine receptors (RyRs). These functional phenotypes due to RGS2 deficiency occur without any alteration in the mechanical properties of uterine arteries. Conversely, uterine arteries deficient in RGS2 hyperconstrict in the absence of a vasoactive GPCR agonist, resulting in marked decrease in wall tension at the expense of decreased lumen diameter at homeostatic perfusion pressure. The effects of RGS2 deficiency on uterine vascular function are likely due to the loss of G protein signaling regulation acting as a brake or a modulator of the development and maintenance of myogenic tone and vascular lumen diameter. Previous reports (Davis and Hill 1999; Holobotovskyy et al. 2015) and findings in this study indicate that control of cytosolic Ca^2+^ by RGS2 through the regulation of G protein signaling is a key mechanism controlling uterine artery tone and blood flow.

Increased cytosolic Ca^2+^ concentration triggers smooth muscle contraction by activating myosin light‐chain phosphorylation and subsequent actomyosin crossbridging that generates contractile force (Zou et al. 2000; Kim et al. 2008; Wang et al. 2009). Activation of G_q_ and G_i/o_ class G proteins in VSMCs stimulates Ca^2+^ release from internal stores via phospholipase C *β* (PLC *β*)‐mediated production of the second messenger, inositol 1,4,5‐trisphosphate (IP_3_), and subsequent opening of sarcoplasmic reticular (SR) IP_3_ receptor channels. PLC *β* activation can also induce Ca^2+^ entry from extracellular source by activating ion channels/transporters in the plasma membrane via the production of diacyl glycerol and activation of protein kinases (Lai et al. 2010). In the intact mammalian circulation, pressure in the circulatory systemic exerts sustained transmural pressure on the vessel wall, and such tonic stimulus in small diameter vessels can be sensed and transduced by G proteins, thereby activating downstream effector proteins, including PLC*β* (Davis and Hill 1999; Coats et al. 2001). Thus, prolonged activation of PLC*β* would be expected to lead to sustained Ca^2+^ release from internal stores required to maintain smooth muscle contraction and increased myogenic tone. Because RGS2 is a potent regulator of G protein signaling in VSMCs, its deficiency likely leads to sustained SR Ca^2+^ release due to prolonged duration of pressure‐induced G protein activation in the vessel wall, resulting in vessel narrowing and decreased blood flow. Several observations in this study support the proposed mechanism. First, uterine artery blood flow velocity is decreased in *Rgs2+/−* and *Rgs2−/−* mice relative to congenic WT control mice. This is accompanied by increases in all derived parameters of vascular impedance to blood flow, including RI, PSV‐to‐LDV ratio, and PI providing further evidence that impaired uterine artery blood flow due to RGS2 deficiency is attributable at least partly to increased vascular tone. Second, myogenic tone at intraluminal pressures equivalent to homeostatic blood pressure, is markedly augmented in uterine arteries from *Rgs2* mutant mice and is normalized to WT level when treated with inhibitors of G_i/o_ and G_q_ class G proteins.

### How does RGS2 deficiency affect intraluminal pressure‐dependent cytosolic Ca^2+^ rise in uterine artery smooth muscle cells?

To address this question, we examined cytosolic Ca^2+^ signaling mechanisms employed by increased intraluminal pressure and vasoactive GPCR stimulation to cause vasoconstriction (Davis and Hill 1999). Although, myogenic constriction does not require agonist‐dependent GPCR stimulation (Davis and Hill 1999; Kauffenstein et al. 2012), increased pressure‐mediated cell stretch is known to activate GPCRs to elicit myogenic response (Mederos y Schnitzler et al. 2008, 2011). Thus, we hypothesized that G protein‐mediated Ca^2+^ signaling mechanisms, as occur in GPCR agonist‐evoked constriction, are central to the development and maintenance of myogenic tone, and that G protein‐mediated cytosolic Ca^2+^ increases, regulated by RGS2, plays a critical role in uterine artery smooth muscle contraction. Consistent with this hypothesis, we found that augmented uterine artery myogenic tone in the absence of RGS2 is abolished by depletion of Ca^2+^ stores. We also found that blockade of Ca^2+^ release from internal stores via RyRs enhances myogenic response in uterine arteries from WT mice to levels equivalent to myogenic tone in arteries from *Rgs2−/−* mice. Moreover, removal of extracellular Ca^2+^ elicits a robust Ca^2+^ release from internal stores via RyRs in isolated uterine artery smooth muscle cells lacking RGS2. Together, these findings suggest that cytosolic Ca^2+^ dishomeostasis due to RGS2 deficiency plays a causal role in the augmentation of uterine artery myogenic tone.

**Figure 7:**
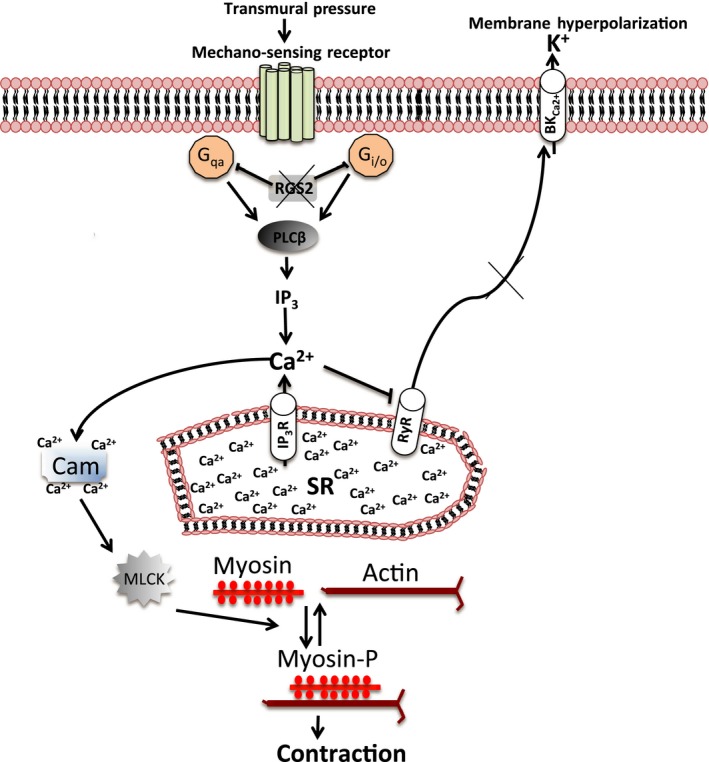
A model summarizing smooth muscle signaling pathways that are regulated by RGS2 to control uterine artery myogenic tone. Under conditions where RGS2 is absent or deficient, G protein activation by mechanosensation leads to a sustained phospholipase C (PLC)‐mediated Ca^2+^ release from the sarcoplasmic reticulum (SR), which increases cytosolic Ca^2+^ concentration and activates Ca^2+^‐Cam/MLCK‐mediated smooth muscle contraction. High cytosolic Ca^2+^ due to the absence/deficiency of RGS2 also inhibits SR Ca^2+^ release via ryanodine receptors (RyR), which normally inhibits contraction by promoting membrane hyperpolarization. Cam, calmodulin; MLCK, myosin light‐chain kinase; BK_C__a_^2+^, Ca^2+^‐activated potassium channel; myosin‐P, phosphorylated myosin.

RGS2 is a well‐established negative regulator of internal Ca^2+^ release via IP_3_ receptor channels following GPCR‐mediated PLC activation (Tang et al. 2003; Heximer 2004; Riddle et al. 2005). Therefore, RGS2 deficiency or absence could lead to a sustained rise in cytosolic Ca^2+^ concentration to levels sufficient to inhibit RyRs (Fill 2003). Accordingly, depletion of cytosolic Ca^2+^ would disinhibit RyRs leading to release from internal stores as observed in uterine artery smooth muscle cells from *Rgs2−/−* mice. Numerous studies in cerebral arteries have shown that internal Ca^2+^ release via RyRs can inhibit myogenic tone by activating Ca^2+^‐activated potassium (BK_Ca_) channels leading to membrane hyperpolarization and inhibition of extracellular Ca^2+^ influx (Nelson et al. 1995; Jaggar et al. 1998; Knot et al. 1998; Wellman and Nelson 2003). Consistent with this mechanism, we found that RyR inactivation increases myogenic tone in WT uterine arteries. Results in this study also suggest that RGS2 acts in this vascular bed to promote RyR‐mediated inhibition of myogenic tone, since ryanodine fails to cause further increase in myogenic tone in arteries lacking RGS2. Moreover, uterine artery smooth muscle cells lacking RGS2 release more Ca^2+^ in the absence of extracellular Ca^2+^, suggesting a higher level of releasable Ca^2+^ pool in internal stores. Whether the increased internal Ca^2+^ pool is due to increased uptake or reduced leakage from SR via RyRs remains to be determined. Taken together, results in this study reveal a novel mechanism by which G protein signaling regulates myogenic tone in uterine arteries (Fig. 7). RGS2 deficiency therefore may cause increased myogenic tone and decreased blood flow due to dysregulation of signaling mechanisms that control cytosolic Ca^2+^ homeostasis.

In conclusion, this study shows that G protein regulation by RGS2 is critical to maintaining normal uterine artery diameter and blood flow. Mutations that lead to decreased RGS2 levels could cause decreased uterine perfusion due to excessive myogenic constriction and increased impedance to blood flow. Therefore, our findings underscore the importance of understanding the physiological and pathophysiological mechanisms that regulate the development and maintenance of myogenic tone. There is growing evidence that RGS2 and other members of the R4 family that are prominently expressed in the vasculature have the potential to regulate smooth muscle function in the uterine vascular bed. Further studies that enhance the understanding of the functions of RGS proteins in this organ system may provide a better insight into vascular mechanisms that are key to achieving normal uterine perfusion, which may lead ultimately to development of a novel treatment or a better diagnosis of uterine artery disorders.

## Conflict of Interest

None declared.
